# Metaheuristic Algorithms Based on Compromise Programming for the Multi-Objective Urban Shipment Problem

**DOI:** 10.3390/e24030388

**Published:** 2022-03-09

**Authors:** Tung Son Ngo, Jafreezal Jaafar, Izzatdin Abdul Aziz, Muhammad Umar Aftab, Hoang Giang Nguyen, Ngoc Anh Bui

**Affiliations:** 1 Department of Computer and Information Sciences, Universiti Teknologi PETRONAS, Seri Iskandar 32610, Malaysia; jafreez@utp.edu.my (J.J.); izzatdin@utp.edu.my (I.A.A.); 2 Information and Communication Department, FPT University, Hanoi 10000, Vietnam; giangnhhe150094@fpt.edu.vn (H.G.N.); anhbn5@fe.edu.vn (N.A.B.); 3 Department of Computer Science, National University of Computer and Emerging Sciences, Chiniot-Faisalabad Campus, Chiniot 35400, Pakistan; ms.umaraftab@yahoo.com

**Keywords:** multi objective optimization, VRP, compromise programming, genetic algorithm, local search, Tabu search, metaheuristics, combinatorial optimization

## Abstract

The Vehicle Routing Problem (VRP) and its variants are found in many fields, especially logistics. In this study, we introduced an adaptive method to a complex VRP. It combines multi-objective optimization and several forms of VRPs with practical requirements for an urban shipment system. The optimizer needs to consider terrain and traffic conditions. The proposed model also considers customers’ expectations and shipper considerations as goals, and a common goal such as transportation cost. We offered compromise programming to approach the multi-objective problem by decomposing the original multi-objective problem into a minimized distance-based problem. We designed a hybrid version of the genetic algorithm with the local search algorithm to solve the proposed problem. We evaluated the effectiveness of the proposed algorithm with the Tabu Search algorithm and the original genetic algorithm on the tested dataset. The results show that our method is an effective decision-making tool for the multi-objective VRP and an effective solver for the new variation of VRP.

## 1. Introduction

### 1.1. Vehicle Routing Problem and Variants

In a logistics system, transportation plays an essential role in moving materials from suppliers to manufacturers, from processing plants to the next step in the production process, or transporting finished products to customers. This scheduling and planning process needs to be calculated before the actual operation. However, this is not an easy task because many resources, such as machines and vehicles, need to be arranged. The problem is called VRP [1]. A capable fleet of vehicles must serve a geographically dispersed group of customers at a minimal cost. We can express the VRP with visitations of the vehicles to customers through a graph as G=(V, E) where $V=\left\{v_{0},v_{1},\ldots ,v_{n}\right\}$ is the set of nodes. $v_{1},\ldots ,v_{n}$ represents the customers to be visited from the depot $v_{0}$. E is an edge set interlinking two locations where E={(i,j)| (i,j)=0,1,…,n, i≠j}. Fundamental decisions are made in the VRP regarding customer assignment to vehicles and the sequence of customers assigned to each vehicle [2]. Many studies on VRP have been conducted, and accordingly, many variants of VRP have also been identified. Some of the more widely known variants include:

- Capacitated Vehicle Routing Problem (C-VRP): refers to the limitation of vehicle capacity for classical VRPs. The system uses multiple vehicles and the total demand of each route does not exceed the vehicle capacity [3,4]. An extension of C-VRP is when the vehicles are heterogeneous (CH-VRP), where each vehicle may have a different capacity [5,6]. The package of products with different types can be considered as the multi product-VRP (MP-VRP) [7].
- Multi-Depot Vehicle Routing Problem (MD-VRP): For the classic VRP problem, the path of all vehicles can only start from a warehouse. In MD-VRP, the vehicles depart from multiple warehouses [7]. Torres et al. reviewed several variants of the MD-VRP, where it can combine several constraints, such as time windows, batch delivery, heterogeneous fleets, and scheduled delivery [8].
- Vehicle Routing Problem with Time Window (VRP-TW): is often encountered in many industrial applications. The time window is divided into soft time windows (delivery not within the period can be penalized) and hard time windows (delivery within the period is mandatory). VRP-TW has received much attention from researchers in recent years [9,10].
- Multi-trip Vehicle Routing Problem (MT-VRP): in MT-VRP, each vehicle is explicitly allowed to perform multiple trips during its service time in such a manner that the total demand of customers served in each route does not exceed the vehicle’s capacity within a given deadline [9,11,12].

VRP is a complex problem that has challenged many researchers. Different variants or business conditions may require the solution search space to be significantly expanded. This paper introduces a method to solve a new VRP that combines multi-objective optimization (MOP) and different forms of VRPs. The proposed solver automatically generates the routings for shippers to deliver packages to urban customers. The urban delivery systems have several characteristics that differ from many ideal environments, in terms of terrain, traffic, and order-warehouse conditions. The scheduler allows consideration of the concern of the business, customer satisfaction, and the employees in the decision-making process. We refer to the problem using the abbreviation MOP-VRP.

### 1.2. Related Works

The VRP problem has many variations. Each business model can potentially become a new variant of the problem, with countless goals and constraints based on the success factors of the business. Table 1 presents some recent studies in this field. In building the optimization model, we can see in these studies that the authors are often only interested in optimizing transportation costs (distance, fuel consumption, transportation cost). The objective functions are usually linear. However, many factors affect this calculation, especially for the case study of transportation in an inner city, such as traffic conditions over time (peak hours) and road conditions (one-/two-way roads). In a previous survey [13], the author noted that the path optimization problem increased the number of real-time path optimization problems considering time-varying factors, such as real-time terrain and real-time traffic conditions involved in MOP-VRP. Therefore, it is a challenge for a simple representation, as used in previous studies, to cover many real-life situations. Several studies related to time windows have made customer satisfaction one of the essential goals. However, in addition to benefiting businesses through cost optimization or satisfying customers, a collaborative economic model requires sharing among stakeholders. Urban transport models often use part-time shippers. If insufficient attention is paid to their needs, the job will become less attractive, and it will not be easy to create a long-term working environment. The lack of committed employees in a volatile business such as logistics results in more difficult constraints to optimize costs and improve service quality. Therefore, these factors need to be considered in the modeling process, and become the objectives to be achieved in the scheduler.

In the decision-making process, decision makers may not be individuals but a group. Therefore, their considerations are critical to any strategies. In real-world business environments, the optimizer needs to pay more attention to multiple decision criteria to meet customers’ requirements. No single solution exists that simultaneously optimizes all objectives of the non-trivial multi-objective scheduling problems. MOP-VRP is not an exception. Several approaches are available to MOP [19] (including MOP-VRP [20,21]). Generally, they are classified into the following: (1) The preference approach, where decision makers have the higher information needed to select the final solution from among several options. (2) The non-preference approach, which assumes that no decision maker is available, or the decision maker does not have the higher information needed to indicate the preferences for the objectives. A user needs only one solution from a practical standpoint, regardless of the optimization problem. In the case of MOP, this creates a dilemma for the user. The ideal MOP procedure requires identifying the trade-off between solutions with a wide range of values for objectives, and then choosing one of the obtained solutions using higher-level information. Significantly, the objective space is higher-dimensional, and it is not easy to visualize the solutions for the users [22]. In this situation, providing an adaptive approach to MOP is a challenge for researchers. The result of solving a real-world problem is usually an approximation set A of the objective vectors (any element of A does not dominate or is not equal to any other accurate vector in A) and not the Pareto optimal front. Okabe et al. reviewed several metrics for accessing the performance of MOP algorithms [23], including cardinality metrics, accuracy metrics, and diversity metrics. Regardless of the approach being used, assessing the quality of the solution is one of the issues that needs to be carefully considered.

Researchers usually model VRPs using binary decision variables to represent relationships between vehicles and destinations, vehicles and depots, etc. These links are expanded exponentially to the number of inputs and may not exist in a solver with a deterministic polynomial time. Therefore, the VRPs are classified into NP-hard and combinatorial optimization. The state-of-the-art approaches presented in the literature comprise two main streams of resolution techniques, namely, exact methods and approximate solution methods (heuristic and meta-heuristic). Exact algorithms provide optimal solutions, and include branch-and-X (bound, cut) [24], dynamic programming [25], and Lagrangian relaxation-based methods [26]. (Meta)heuristics include simulated annealing and population-based methods, such as the evolutionary algorithm [27], and generally yield near-optimal solutions. The exact methods are more suitable for problems that having a small size. However, logistic systems are increasingly used at larger scales, and a greater number of orders, customers, and vehicles, etc. Hence, (meta)heuristics is a better choice due to its flexible search capabilities and easy integration for exploiting the good properties of different methods.

Many researchers have designed heuristic and metaheuristic algorithms, and combinations of VRP and its variants. Samuel Nucamendi-Guillén (2021) [16] developed a metaheuristic procedure to find a solution by improving the initial solution using local search algorithms. Zhiwei Liu (2017) [28] proposed a method that combines Tabu with mem-brane computing to find the solution for VRPTW. Babaee Tirkolaee E (2019) [10] developed simulated annealing (SA), a local search algorithm that can escape the local optimum for MT-VRPTW in urban waste collection. To solve the MT time-dependent VRPTW. Binbin Pan [13] designed a hybrid metaheuristic algorithm using variable neighborhood descent (VND) for intensive exploitation and adaptive extensive neighborhood search (ALNS) to direct the inquiry when VND is stuck in a local optimum.

Among several branches of metaheuristics, evolutionary algorithms (EAs) have attracted the attention of many researchers. For instance, Hari Kurnia (2018) [29], Cortes (2018) [30], and R Fitriana (2019) [31] designed a classical genetic algorithm for VRP, CVRP, and MDVRP, respectively. Regarding VRP with more features and attributes that reflect the complexity of the real problem, a hybrid genetic algorithm that improves the solution by implementing a local search heuristic in the initial phase of the genetic algorithm was proposed by Rabbouch (2019) [32]. Jalel Euchi (2021) [33] solved another complex VRP involving drones using a modified hybrid genetic algorithm combined with a nearest neighbor heuristic, and modifying the saving heuristic in the initial phase. Although the nearest neighbor heuristic helps improve the initial solutions, the saving heuristic prevented the genetic algorithm from falling into an early local optimum. Yanfang Ma (2017) [14] proposed an improved ant colony optimization (ACO) combined with the nearest neighbor search method for the MD VRPTW. Wei-heng Zhang (2020) [15] designed a two-stage ACO for MDGVRP, assigning customers to the depot to generate routes. EAs and their hybrid versions have been proven to be effective for single objective VRPs. They can obtain a set of solutions present in a solution process, provide the ability to be easily determined with different types of variables, and do not require any assumptions that make convexity and separability distinctions between the objectives and related constraints. In general, these algorithms provide a design direction. The suggested search operations are based on different designer arguments. In general, many factors determine the extent this problem. However, in our opinion, building a suitable data structure plays a vital role. A good data structure can support stochastic operations and help improve population diversity, while ensuring the algorithm’s convergence. Multi-Objective EA (MOEA) extends EAs to deal with multi-objective optimization problems. They can be classified based on different features. A widely accepted classification for MOEA considers Pareto-dominance-based, decomposition-based, and indicator-based algorithms [34]. MOEAs have been applied in several applications [35,36] that can search for a set of optimal solutions on the Pareto front. However, this involves much higher-level information, which is often non-technical, qualitative, and experience-driven, to indicate the final solution, with a prohibitive computational cost. This cost is not suitable in many experimental conditions. Deriving an efficient approach for MOP-VRP that does not require pre-determination of the trade-off between objectives, and can be integrated with the algorithm design to maintain the solution quality with a reasonable cost, has always been a challenge in this field [37].

### 1.3. Contributions

This study presents an adaptive method for a variant of MOP-VRP as a scheduler in an urban delivery system. The model is built around the real-life requirement of the urban delivery system. The optimizer needs to provide the solution to satisfy multiple business conditions that comply with essential factors, such as terrain and traffic conditions, in addition to other constraints for VRP. We use compromise programming to approach the proposed MOP. This allows decision makers to obtain an optimal solution without defining preferences on each objective function in advance. However, if they do, alternative decision strategies are still used generally through the definition of weights to assign the effect of objective functions via the distance function. We designed and compared Tabu search (TA), the genetic algorithm (GA), and a combination of GA and the local search algorithm (HGA) to solve the proposed model on the tested dataset. Our study suggests a new variant of VRP. This can benefit researchers and engineers to develop a better optimizer for variants of VRP. This research also contributes to the developed methodology for multi-objective scheduling and planning problems [38]. The remainder of this paper is organized as follows. The proposed model and algorithm are respectively described in Section 2 and Section 3. To evaluate the proposed approach, we present the experiments and discussion in Section 4. Finally, Section 5 offers a conclusion.

## 2. Proposed Model

In this study, we built a multi-objective optimization solver for the urban shipment problem. This section describes the mathematical optimization model and the approach used in the proposed multi-objective optimization problem. The goals to be achieved by the developed scheduler were thoroughly discussed with logistic managers as the decision makers. Some important business rules were defined as follows:

- The system is set up with many delivery points corresponding to the customers.
- Packages that need to be delivered have different weights and delivery periods.
- Packages are shipped from various warehouses.
- Each package needs to be collected from the allocated warehouse.
- The delivery time between any two locations is time- and terrain-dependent. It is estimated based on statistical data from previous shipments.
- Shippers also use vehicles with different payloads and transportation costs.
- To deliver the order, the shipper may need to take several trips.

### 2.1. Mathematical Formulation

**Figure 1:**
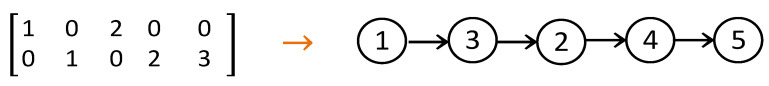
An example of a planned path with $N=5,\;V_{k}=2$.

The decision variables represent the whole detailed plan to shippers. In addition, several dependent variables that are computed from the decision variables were also introduced in the model, as follows:

- C represents the set of delivery points/customers.
- K denotes the set of shippers.
- D represents the set of warehouses.
- $N=D\;\cup^{\;}\;C$ denotes the set of locations, where the first |D| elements are the locations of the depots, and the last |C| elements represent the locations of customers.
- $B\;\in \;{\mathbb{R}}^{\left|K\right|}$ is the vector that represents the capacity of shippers, where $B_{k}\;\in {\mathbb{N}}^{*}$ is the capacity of shipper kth.
- $P\;\in \;{\mathbb{R}}^{\left|K\right|}$ is the vector that represent the freight rates of the shippers, where $P_{k}\;\in R^{*}$ is the freight rate of shipper kth.
- $W\;\in \;{\mathbb{R}}^{\left|C\right|}\;$ is the vector used to illustrate the weight of the orders by customers, where $W_{c}\in {\mathbb{N}}^{*}$ is the weight needed to deliver to delivery point cth.
- $L\;\in \;{\mathbb{R}}^{\left|C\right|}\;$ is the vector used to illustrate the load time of the orders by customers, where $L_{c}\in {\mathbb{N}}^{*}$ is the time needed to load the package of customer cth.
- A$=\left\{A^{c}|A^{c}\in {\mathbb{R}}^{2},c\in C\right\}$ is the vector that represents the appointment time of the customer, where [$A_{\mathrm{soon}}^{c},A_{\mathrm{late}}^{c}\;]$ respectively describes the appointment time and the time window that customer cth demands for his/her order.
- $M=\left\{M^{k}|M^{k}\in {\mathbb{R}}^{\left|N\right|\times \left|N\right|},k\in K\right\}$, $M^{k}=\left\{M_{i,j}^{k}|M_{i,j}^{k}\in {\mathbb{N}}^{*},\;i,j=1\ldots \left|N\right|\right\}$ where $M_{i,j}^{k}$ is the distance if shipper k goes from location ith to location jth.
- $T=\left\{T^{k}|T^{k}\in {\mathbb{R}}^{\left|N\right|\times \left|N\right|},k\in K\right\}$,$\;T^{k}=\{T_{i,j}^{k}|T_{i,j}^{k}\in {\mathbb{N}}^{*},\;i,j=1\ldots \left|N\right|\}$ denotes the time consumption of transportations between locations for shipper k, where $T_{i,j}^{k}$ represents the time that shipper kth takes to travel from location ith to location jth. $T_{i,j}^{k}$ is computed based on time(i,j,k,t), which is the function to query the traveling time of shipper kth from location ith to location jth, where t represents the start time. The return value is depended on the traffic condition at time t.
- $U\in {\mathbb{R}}^{\left|C\right|\times \left|D\right|}$ is a matrix to represent the links of the warehouse that stores the orders and the delivery points. $U_{c,d}=1$ means the order of customer cth is kept by warehouse dth.
- $V=\left\{V_{k}|V_{k}\;\in {\mathbb{N}}^{*},k\in K\right\}\;$ is the vector that represents the number of tours each shipper takes, where $V_{k}$ is the number of tours of shipper  kth.
- The decision variable $O=\{O^{k}|O^{k}\in \;{\mathbb{R}}^{V_{k}\times \left|N\right|},\;k\in K\}$ represents the planned paths for shippers, where $V_{k}\;\in \mathbb{N}$ denotes the number of tours made by the shipper kth to deliver all of his/her assigned orders. Figure 1 illustrates an example of a planned path.

- $Z=\{Z_{k,v,i,j}|Z_{k,v,i,j}=equal\left(\left\{\min(O_{v,j}^{k},1)-\min(O_{v,i}^{k},1)\;,\;1\right\}\right),\;\forall \;k=1\ldots \left|K\right|,\;i=1\ldots \left|N\right|,\;j=1\ldots \left|N\right|,v=1\ldots V_{k}\;\wedge Z_{k,v,S_{v}^{k},0}=1\;\}$ represents the sequence of visited nodes of the shippers. Shipper kth goes to node ith immediately after node jth in trip vth if $Z_{k,v,i,j}=1$. 0 otherwise.
- $S=\{S_{c}|S_{c}\in {\mathbb{R}}^{*},\;c\;\in C\}$ where $S_{c}$ is the estimated time to deliver to the customer c. If $S_{c}$<$\;A_{soon}^{c}$ then $S_{c}=A_{soon}^{c}$, and the duration $A_{soon}^{c}-S_{c}\;$is considered to be waiting time.
- $Y=\{Y_{k}|Y_{k}\in {\mathbb{R}}^{*},\;k=1\ldots K\;\}\;$ where $Y_{k}=\overset{V_{k}}{\underset{v=1}{\sum }}\overset{\left|N\right|}{\underset{i=1}{\sum }}\overset{\left|N\right|}{\underset{j=1}{\sum }}Z_{k,v,i,j}*T_{i,j}^{k}$ represents the total traveling time of the shipper kth.

To meet business requirements, the solver must satisfy the following objectives:

- Minimize of the transportation cost of all shippers based on types of vehicles. Traveling on long routes increases costs:

$$\min\;\left(f_{1}\left(O\right)=\overset{\left|K\right|}{\underset{k=1}{\sum }}\overset{V_{k}}{\underset{v=1}{\sum }}\overset{\left|N\right|}{\underset{i=1}{\sum }}\overset{\left|N\right|}{\underset{j=1}{\sum }}Z_{k,v,i,j}*P_{k}*M_{j,i}^{k}\right)$$

- The urban delivery requires punctuality, although this is not a hard constraint on the model. However, the less late the delivery, the more satisfied the customer. The optimizer needs to minimize late delivery to the customers:

$$\min\;\left(f_{2}\left(O\right)=\overset{\left|C\right|}{\underset{c=1}{\sum }}late\left(S_{c}\right)\right)$$

where: $late\left(S_{c}\right)=\{\begin{array}{c} 0\;if\;A_{late}^{c}\geq \;S_{c}\;\\ S_{c}-A_{late}^{c}\;if\;S_{c}>A_{late}^{c} \end{array}$

- Serving customers is beneficial for businesses. However, it can be traded off by the convenience of the delivery staff. The workforce is usually part-time. Thus, a route that saves shippers waiting time provides a competitive environment. It is necessary to minimize the waiting time of the shippers:

$$\min\;\left(f_{3}\left(O\right)=\overset{\left|C\right|}{\underset{c=1}{\sum }}wait\left(S_{c}\right)\right)$$

where: $wait\left(S_{c}\right)=\{\begin{array}{c} 0\;if\;A_{soon}^{c}\leq \;S_{c}\;\\ A_{soon}^{c}-S_{c}\;if\;S_{c}<A_{soon}^{c} \end{array}$

- Minimize differences in traveling time of the shippers. The shipper’s working time is only counted as travel time. This does not include waiting time. Therefore, this time allocation helps to balance the workload of the shippers:

$$\min\;\left(f_{4}\left(O\right)=\overset{\left|K\right|}{\underset{k=1}{\sum }}\left(\left|Y_{k}-\frac{1}{\left|K\right|}\overset{\left|K\right|}{\underset{i=1}{\sum }}Y_{i}\right|\right)\right)$$

Subject to:

- All orders must be delivered:

$$\overset{\left|K\right|}{\underset{k=1}{\sum }}\overset{\left|N\right|}{\underset{i=\left|D\right|+1}{\sum }}\overset{V_{k}}{\underset{v=1}{\sum }}\min\left(O_{v,i}^{k},1\right)=\left|C\right|$$

- Each delivery point is assigned to only one shipper:

$$\overset{\left|K\right|}{\underset{k=1}{\sum }}\overset{V_{k}}{\underset{v=1}{\sum }}\min\left(O_{v,i}^{k},1\right)=1\;\forall i=\left|D\right|+1\ldots \left|N\right|$$

- The capacity of the shipper on every trip is respected:

$$\overset{\left|C\right|}{\underset{c=1}{\sum }}\min\left(O_{v,c}^{k},1\right)*W_{c}\leq B_{k}\;\forall k=1\ldots K,\;v=1\ldots V_{k}$$

- The shipper must load the customer’s package before delivery to the customer in the same trip:

$$O_{v,c}^{k}-O_{v,d}^{k}\geq U_{c,d}\;\forall k=1\ldots \left|K\right|,\;v=1\ldots V_{k},\;c=\left|D\right|+1\ldots \left|N\right|,\;d=1\ldots \left|D\right|$$

- The shipper cannot visit more than one location at the same time:

$$O_{v,i}^{k}\neq O_{v,j}^{k}\;\forall k=1\ldots K,\;v=1\ldots V_{k},\;i=1\ldots \left|N\right|,\;j=1\ldots \left|N\right|,i\neq j,O_{v,i}^{k}\neq 0\;,\;O_{v,j}^{k}\;\neq 0$$

### 2.2. Compromise Programming for MOP-VRP

**Figure 2:**
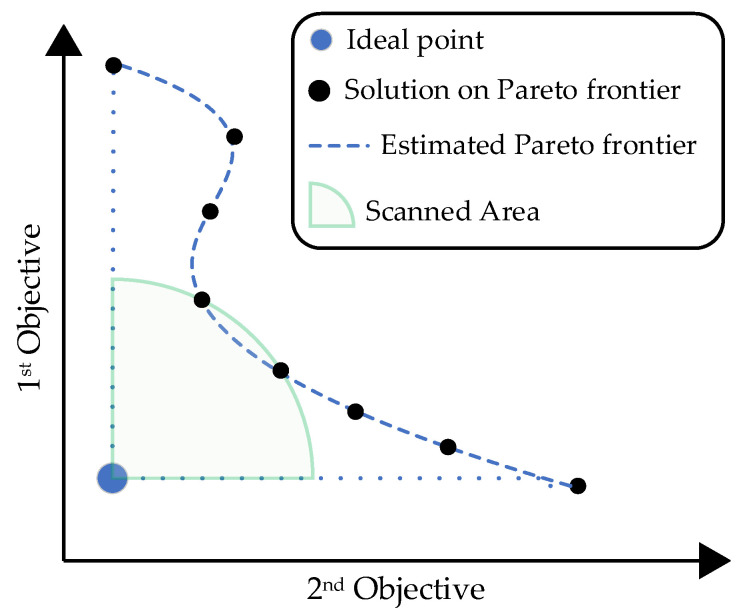
Scanned area of the search process in the CP-based approach.

Compromise programming (CP) [39] is based on the idea of not using any preference information or relying on assumptions about the importance of objectives. The method does not try to find multiple Pareto optimal solutions. Instead, the distance between some reference point and the feasible objective region is minimized to find a single optimal solution, as shown in Figure 2. For this purpose, the weighted $L_{p}$ metrics measure the distance of any solution from the reference point. The ideal objective vector is often used as the reference point:

$$\min{\left(\overset{N}{\underset{i=1}{\sum }}w_{i}{\left|f_{i}\left(x\right)-z_{i}^{*}\right|}^{p}\right)}^{1/p}\;s.t.\;x\in X$$

where *x* is the decision variable and *X* is the feasible set, $z_{i}^{*}=\underset{x\in X}{\min}f_{i}\left(x\right)$, p can take any value between 1 and ∞ (in practice normally p=2), the weight vector $w=\text{\{}w_{i}|w_{i}\;\in \;{\mathbb{R}}^{+}\;i=1\ldots N\}$, and N is the number of objective functions. The literature suggests normalizing the dimensional values in the range [0,1] of the distance function. We can rewrite the objective function in the form of norm 2 as: $\min{\left(\sum_{i=1}^{N}w_{i}{\left|\frac{F_{i}-z_{i}^{*}}{\;z_{i}^{worst}-z_{i}^{*}}\right|}^{2}\right)}^{1/2}$, where $\;z_{i}^{worst}=\underset{x\in X}{\max}f_{i}\left(x\right)$.

Many studies have used CP to approach the MOP problem, such as for university timetabling [40,41,42], team selection [43], in a knowledge-based recommender [44], and project task assignment [45]. However, this t may require pre-defined minimal and maximal values of the objective functions. Although some of these values are predictable [43], most other cases require the problem to be solved as a single objective function multiple times, which may be costly. Other studies [44,46] show that the referential point may be selected from business estimations, which can provide better performance for the agents in the searching process. However, in this study, the normalization method using both of $\;z_{}^{*}$ and $\;z_{}^{worst}$ resulted in a solution having better quality.

## 3. Proposed Algorithms

In this section, we introduce the proposed algorithms. The main algorithm is HGA, which combines GA and local search. The first part of this section describes GA. We use the same principle for the search agent’s stochastic process and data structure for proposed algorithms. The algorithms described after GA share several common strategies. The second part describes how we implement HGA. Another algorithm, TA, which does not belong to the class of evolutionary algorithm, is also proposed to evaluate the approach’s effectiveness.

### 3.1. Genetic Algorithm

**Figure 3:**
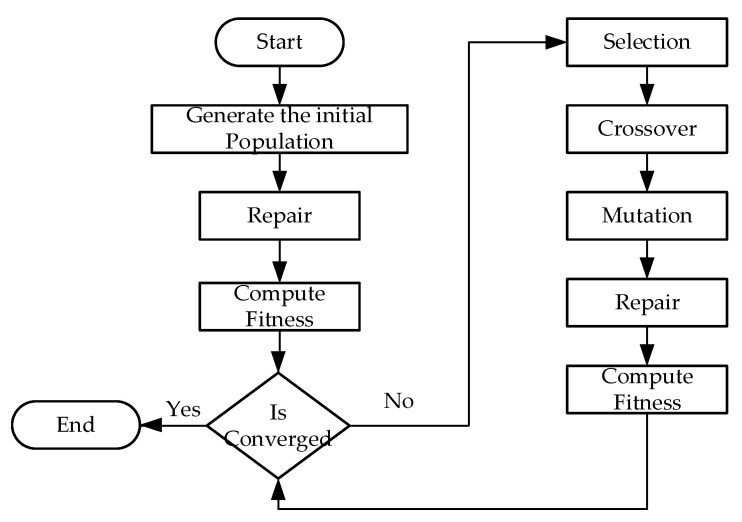
The flow of the proposed GA scheme.

GA is one of the most well-known metaheuristic algorithms used to solve NP-hard problems and belongs to the family of evolution algorithms [47]. The process of natural evolution is the inspiration for the idea of GA. The algorithm begins with a random population, in which each individual represents a solution to the problem. The final solution is obtained through the evolution of the population. The designed scheme of GA is shown in Figure 3. The fundamental difference between our design and the traditional flow is that we introduce a repair step to fix instances that violate the constraints during the random process. To initialize the first population, we randomly create the route for each solution of the initial population. With an initial route, we obtain the list of assigned customers for each solution, and then sort them by their demand time in ascending order. After modifying the route, the trip is created using the idea that shippers only need to return to the depot if their job is finished or the trip’s capacity is overloaded. They then need to return to the depot.

**Figure 4:**
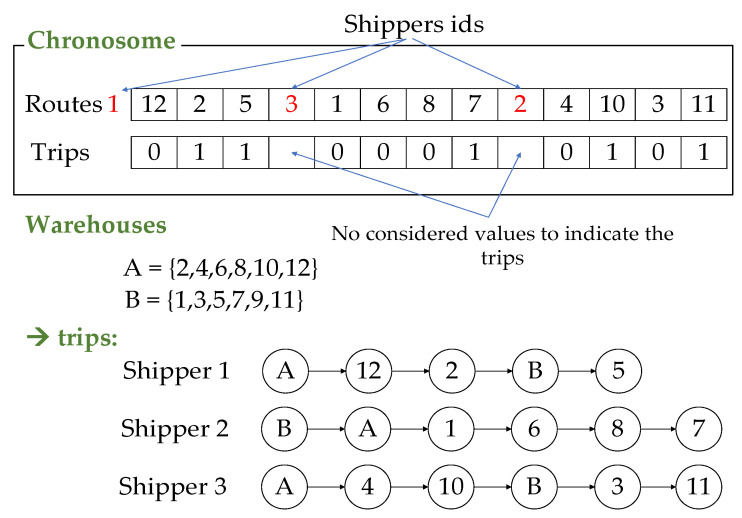
Chromosome representation.

**Figure 5:**
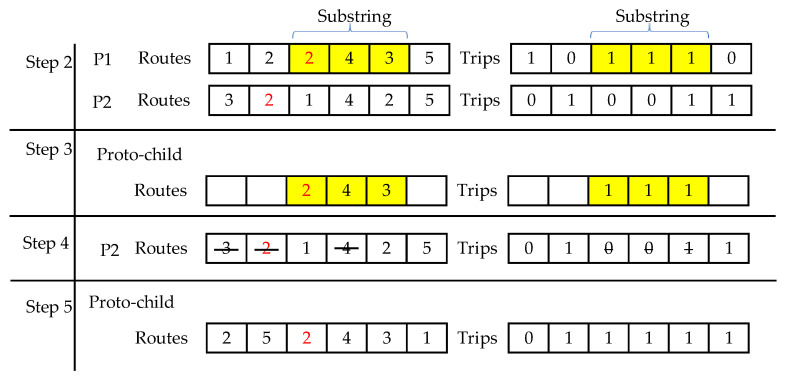
Step 2 to step 5 of the crossover phase.

1. Initialize the population: The structure of the individual is equivalent to decision variable O, as described in the proposed optimization model. We generate the population P as the set of π individuals. For programming convenience, the chromosome is represented by two arrays having the size of (C+K−1), denoted by routes and trips. routes represents the paths of the shippers by storing the identifications of C customers and (K−1) shippers, and arranging them in random order. Positive integers are used to represent the customer ids and negative integers are used to represent the shipper ids. trips is used to identify the trips of the shippers. Figure 4 shows the chromosome representation for an example of 12 customers with ids from 1 to 12, three shippers with ids 1 to 3, and two warehouses A and B. In the figure, the first three elements of routes are 12, 2, and 5. This means the shipper with id 1 is assigned to deliver to these customers. The corresponding elements in trips are binary only. A value of 0 means the shipper can directly continue to travel, whereas 1 means the shipper has to return to the related warehouse to load the package before delivering to the next customer. trips stores only K−1 values; in this case, it is not necessary that shipper id 1 is stored in routes, thus increasing the convenience of the random process.
2. Fitness function: we used the compromise Euclidean distance-based function for the individual as:
  $$p.fitness={\left(\overset{4}{\underset{i=1}{\sum }}w_{i}{\left|\frac{F_{i}-z_{i}^{*}}{\;z_{i}^{worst}-z_{i}^{*}}\right|}^{2}\right)}^{1/2}\;\forall \;p\;\in P$$
  All proposed algorithms give optimal results for single-objective optimization problems. $\;z_{i}^{*}$and $\;z_{i}^{worst}$ can be considered as pre-computed in this procedure.
3. Selection: we chose φ elite individuals and bypassed them from the crossover and mutation phase to keep them in the next generation.
4. Crossover: creates a new solution that retains the good properties of its parent. We selected a crossover rate μ. There are five steps to implement the crossover for the remaining individuals of the next generation (see Figure 5), as follows:
  Step 1: Randomly select two individuals as the parents denoted by $p_{1},p_{2}$
  Step 2: Randomly select a substring from a parent for both routes and trips.
  Step 3: Create a proto-child by phasing the substring into its corresponding position.
  Step 4: Delete all the elements that are already in the proto-child of the remaining parents. This creates an array that contains the elements needed by the proto-child.
  Step 5:
  ▪ For routes: Place the elements of the resulting array into the unfix position of the proto-child from left to right.
  For trips: Place the elements of the resulting array into the unfix position of the proto-child in the corresponding position.
5. Mutation: Modify a solution to create a new solution to expand the search space of the algorithm. We selected a mutation rate ω. There are two steps to implement the mutation for the remaining individuals of the next generation, as follows:
  Step 1: Randomly select a substring from the individual.
  Step 2:
  ▪ For routes: shuffle the element in the substring to create a new route.
  For trips: flip each element in the substring to create a new trip.
6. Repair: In this phase we fix the solutions that violate the defined constraints. There are some principal rules to guide the repair process:
  ▪ The trips array controls the trips of the shippers that are related to the capacity. If the customers’ weight is already surpassed for the corresponding shipper in a journey, the trips array must be fixed for that shipper to return for supply after the current customer.
  We maintain the principal to minimize the number of trips; therefore, we check *trips* to determine if any trips[i]=1 can be removed without violating the capacity constraint, and remove these if possible.
  At the end of each trip, the corresponding element value in the trips array must be 1.
  trips[i]=1∀ i=1…(C+K−1) ∧routes[i]<0

### 3.2. Hybrid Genetic Algorithm

#### 3.2.1. Local Search

Local search [48] is an algorithm using a single search path (searching in the neighborhood) to improve the initial solution and thus achieve a better solution. The solution point is structured in the same manner as for the chromosome representation of GA, as described in Section 3.1. The process of the local search algorithm can be described in two steps as follows:

1. Denote s as the starting solution.
2. Find S=searchNeighborhood(s,k), which is the set of neighboring solutions of s.

where: k is the size of S.

searchNeighborhood(s,k) is a function to return the k neighbor’s solutions.

3. Repair every solution $s^{\prime }$ in S that violates the defined constraints.
4. Return s* = $\underset{s^{\prime }\in S}{\mathrm{argmax}}\left(s^{\prime }.fitness\right)$.

#### 3.2.2. Combination of GA and Local Search

**Figure 6:**
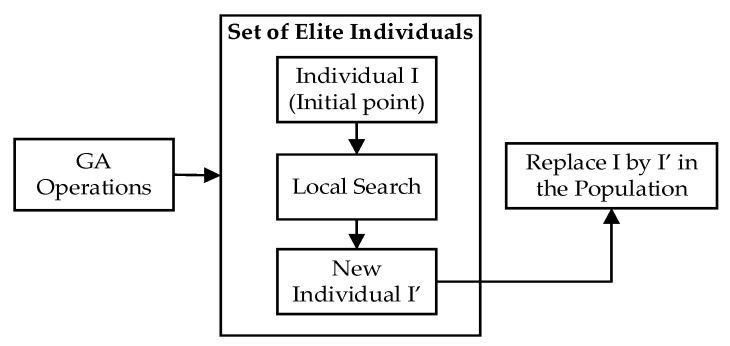
Combination of GA at the gth generation and local search in HGA.

One risk associated with GA is that individuals can become trapped in local optima, often caused by designs that fail to maintain population diversity or an insufficient number of search agents. In this study, we take advantage of the better neighborhood search feature of local search to give individuals a better chance of overcoming the problem of being stuck in a local optima. We run the local search algorithm several times, corresponding to the elite individuals obtained by GA as starting points to retrieve better solutions. These solutions in the next generation then replace the inputs. The process is illustrated in Figure 6.

### 3.3. Tabu Search

**Figure 7:**
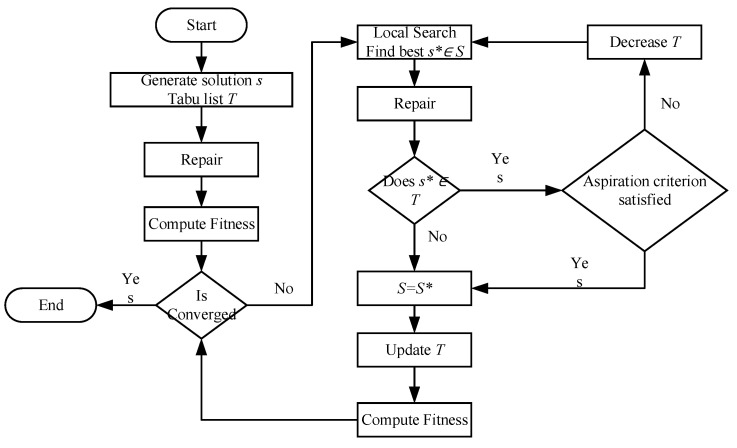
The flow of TA.

Tabu search is an improved version of local search used for mathematical optimization [49]. Local search methods tend to become stuck in suboptimal regions. TA enhances the performance of these techniques by banning accessed solutions or other solutions through user-supplied rules. We implement the principal mechanism of TA and reuse the data structure and algorithms described in the previous sections. The flow of TA is illustrated in Figure 7.

## 4. Experimental Results

### 4.1. Experimental Design

**Figure 8:**
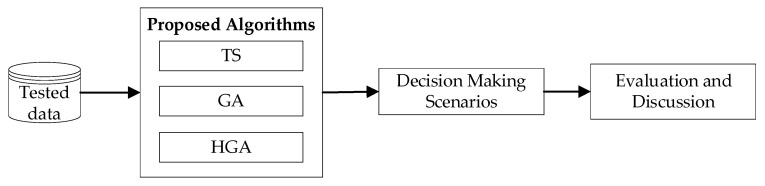
Overview of the experimental design.

To evaluate the effectiveness of the proposed method, we used a collected dataset for a single business day received from a shipment company in Hanoi, Vietnam. This consisted of 200 orders, distributed from five warehouses, and delivered by ten shippers. Customer locations were collected via GPS. To avoid detailed measurements in the scheduling process, the company transformed the customer’s precise coordinates to the center of the street. The travel time and the average speed of shippers at a given time were measured based on estimation of the check-in data of the shippers. Figure 8 illustrates the overview of the experimental design.

We conducted experiments and analyzed the results of the three proposed algorithms, namely, TA, GA, and HGA, in terms of convergence, processing speed, and solution quality. Then, the best-performing algorithm was selected for testing with different decision scenarios. The experiments were implemented in a computer having the configuration shown in Table 2.

Metaheuristic algorithms are governed by parameters. We tested several different parameter values. Each of these can affect both the computational cost and the quality of the solution. For example, the more search agents that are used, the greater the chance of finding the global optima. However, the search time of each agent is also increased, which significantly increases the computation time. The experiments were performed with the appropriate settings to highlight the performance of each designed algorithm, as shown in Table 3.

### 4.2. Results

As mentioned in Section 2.2, the original objective functions were transformed to a distance function using compromise programming. We solved the problem as separate single-objective problems. The worst point was identified in the same manner as the ideal point, but with the use of the max function for the objective function. The ideal point and worst point are shown in Table 4. The three designed algorithms gave the same result when solving these single-objective problems.

**Figure 9:**
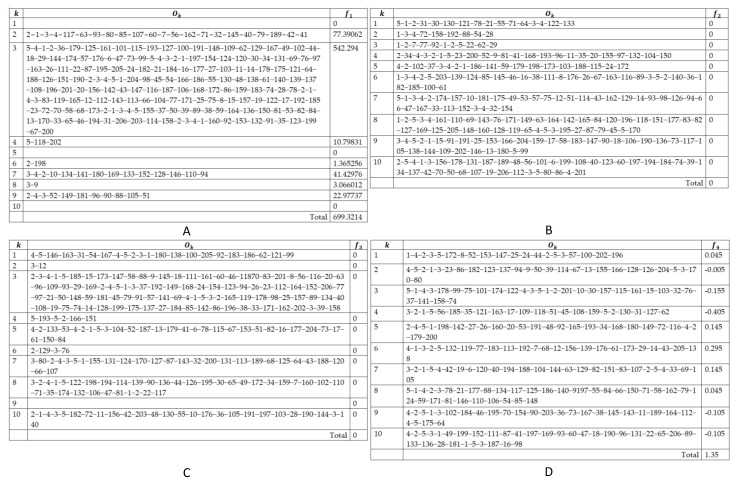
Generated traveling paths for shippers by solving single-objective problems: (**A**) $f_{1}$; (**B**) $f_{2}$; (**C**) $f_{3}$; (**D**) $f_{4}$.

The detailed solution of each single-objective problem is described in Figure 9. In the first case, we aimed to achieve the lowest transport costs. The system only needs to use seven of 10 shippers, as shown in Figure 9A. However, the late delivery time is considerable (181,801.5), and the time the shippers wait and the difference in workload are 2705.85 and 2274.8 time-units, respectively. All shippers are mobilized to deliver on time (Figure 9B). However, the transport cost also increased to 2438.09. To avoid late delivery, shippers must arrive earlier than the scheduled time for several orders, then wait until the right time to deliver. The total waiting time is 6894.85. The difference in workload is also relatively significant when the shipper with the highest workload has to work 257,875 time-units more than the average. Figure 9C,D show the results when the scheduler optimizes the objectives f3 and f4 as, respectively, $\left(f_{1}=2676.491;f_{2}=\text{152,876.1};f_{3}=0;f_{4}=2649.96\right)$ and $\left(f_{1}=2734.456018;f_{2}=\text{99,640.8};f_{3}=6635.5;f_{4}=1.35\right)$.

To compare the algorithms, we set the weight parameters to be the same, although, theoretically, multi-objective optimization may not yield the best solution. When a solution did not entirely dominate other solutions, we ranked the solutions based on the obtained values of the distance function (objective values). The metaheuristics operations are stochastic. Therefore, to evaluate the stability of the proposed algorithms, we executed them 15 times; the obtained results are shown in Table 5. The numbers show that GA-based algorithms can receive quality results with a much smaller cost than TA. Using multiple search agents in TA, each of which continues to search for quality neighbors, is a computationally expensive process. To achieve a similar solution quality as that of HGA, the average processing time of TA is 2.06 times greater on the tested dataset. We normalized the objective values in the range [0, 1] based on the obtained values in Table 4. We only used a single core to execute the algorithms. The time used for execution can be reduced by using the parallel mechanism for search agents proposed by Ngo et al. [44].

Both GA and HGA algorithms use a similar search mechanism. The only difference is that HGA continues to use local search to find neighbors with better fitness values before creating a new generation. Theoretically, this ensures that the HGA has a better chance of avoiding the local optima than the original version. This was also confirmed in our experiments. However, because many individuals must perform local search operations after genetic operations, the total time required to search for each solution increases significantly. Nonetheless, HGA can provide high-quality solutions when the obtained solution completely dominates the solutions of the original GA, and was slightly better than TA on the tested dataset of 200 customers.

**Figure 10:**
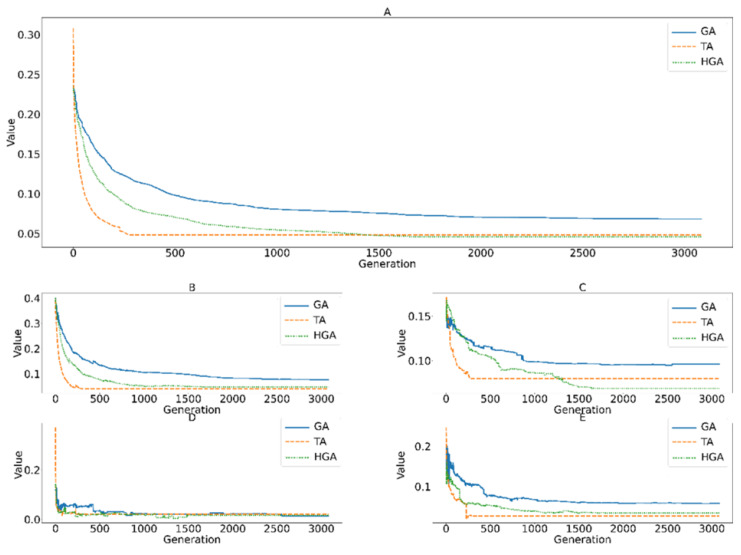
(**A**) Change in the fitness values; (**B**) $\;f_{1}\left(O\right)$; (**C**) $\;f_{2}\left(O\right)$; (**D**) $f_{3}\left(O\right)$; (**E**) $f_{4}\left(O\right)$ of the designed algorithms over generations/iterations.

The change in fitness values can be used to visualize the convergence of the algorithms through each generation/iteration, as shown in Figure 10. For convenience, the figure shows a comparison of all algorithms running up to 3000 iterations. However, these algorithms still respected their stop conditions. The result mentioned in the previous section is the time taken to reach the final solution. The change in fitness values shows that TA obtained better results in the first few iterations than the GA-based algorithms. However, up to the 297th iteration, the fitness value of 0.048 is a local solution that TA cannot pass. By comparison, the GA and HGA algorithms show that they maintained the population diversity because the next generations continue to improve the quality of the solution. HGA provided solutions that were continuously improved over the generations, until finding the final solution (0.046) at the 1849th generation. This continuous improvement is significant in practice. The algorithmic stopping is a minor condition that can be determined by the number of generations with the same result to avoid an increase in the computational cost. The different objective functions may increase in some generations, but they decrease in general because the algorithm consistently reduces the fitness values.

**Figure 11:**
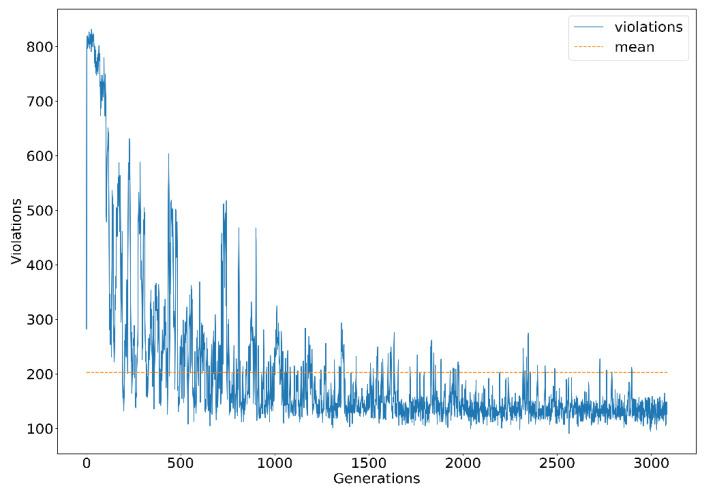
Number of violated constraints with corresponding iterations of the search process of HGA.

The stochastic mechanism for generating solutions generates a series of solutions that violate constraints. In some cases [45], these solutions can be eliminated by the searching process. A mechanism must be used to correct the error solutions in this problem. This process reduces the time taken by the algorithm through the acquisition of valid answers. The number of invalid solutions decreases after newly generated solutions. For example, for the GA-based algorithms, parent solution genes that do not violate the constraints are selected and crossed. However, the mutation process produces a certain number of invalid solutions. Figure 11 displays the number of violated constraints with the corresponding iterations of the search process of HGA. The data distribution affects the reduction in the values in the distance-based fitness function. For example, the value of objective function $f_{4}$, the workload of the shipper, seems to have played a more significant role than the dense distribution of the values in the objective function $f_{1}$, as observed from the solution generated by HGA in Table 6. However, the search operations can be directed by calibrating the weight parameters.

**Figure 12:**
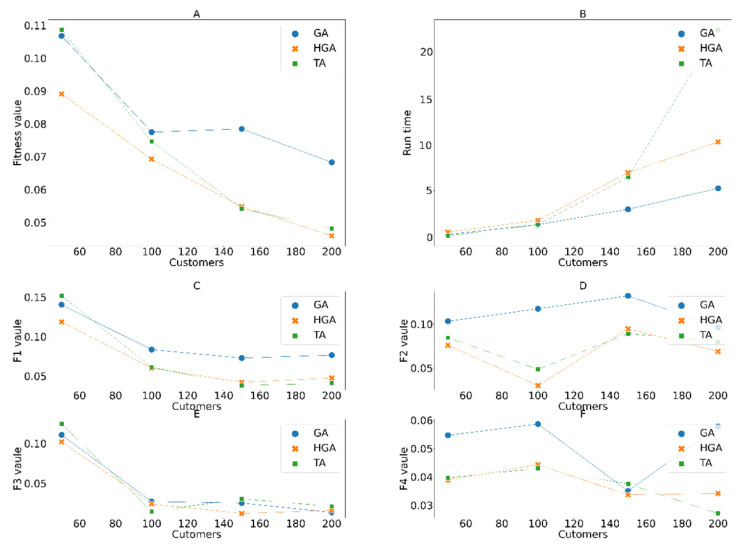
(**A**) Fitness values; (**B**) execution time; (**C**) $f_{1}$; (**D**) $f_{2}$; (**E**) $f_{3}$; (**F**) $f_{4}$; obtained with different numbers of customers to serve.

To evaluate the adaptability of the algorithms to different scales of the system, we divided the test dataset into smaller datasets having 50, 100, 150, and 200 customers, respectively, to conduct the experiments, as shown in Figure 12. The processing speed of TA slows in proportion to the system scale. The quality of HGA is slightly better than that of TA and significantly better compared to that of the original GA. The processing time of HGA increased more quickly than that of GA but was better than that of TA. HGA and TA both use neighbor searching, but genetic operations seem to be more effective at identifying initial points before searching for neighboring points than TA’s hill-climbing mechanism.

**Figure 13:**
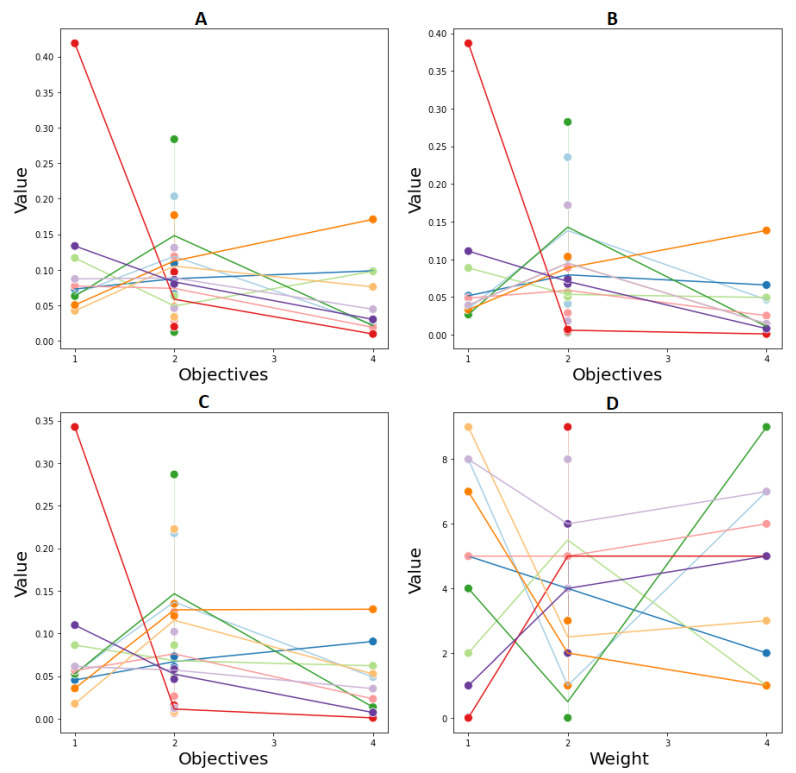
Ten obtained solutions in 4D objective space by (**A**) GA, (**B**) TA, (**C**) HGA with different weight parameters (**D**).

Approaches to the MOP problem based on the decomposition of multi-objective functions to single-objective functions have many advantages. Compromise programming is a suitable solution when the decision maker cannot assign preferences for each specific goal. However, its disadvantage is that is very difficult to illustrate the Pareto frontier. Nonetheless, using weight parameters, decision makers can experiment with different decision criteria. We compared the solutions generated by the proposed algorithms. These solutions do not fully dominate (all objective values are better) each other. Therefore, to evaluate which algorithm performs better in different decision-making situations, in this experiment, we selected the sub-dataset of 100 customers then obtained ten solution points corresponding to different values of weight parameters for each algorithm, as shown in Figure 13. We then calculated the hypervolume HVC [50] for the solutions obtained by the algorithm as follows:

$$HVC=\frac{volume\left(\cup_{s\in S}\left(s,z^{worst}\right)\right)}{volume\left(cube\left(z^{*},z^{worst}\right)\right)}$$

where:

- s  is the solution in the Pareto solution set S that is generated by the algorithm.
- cube(a,b) denotes the oriented axes hypercube that is formulated by points a and b in the objective space.
- volume(c) denotes the volume of the hypercube c in the objective space.

The results listed in Table 7 show that the HGA’s hypervolume is similar to that of TA and better than that of the GA. The larger the HVC value, the closer the algorithm can discover solutions close to the actual Pareto frontier. Through TA’s nearest neighbor search, the hill-climbing mechanism allows it to overcome the local optimal better than the original GA. However, GA can be effectively integrated with other methods to improve quality without incurring significant computational costs. The hybrid version of EA shows its effectiveness in different decision-making scenarios.

To evaluate the capabilities of the proposed CP-based method, we used genetic operations designed to implement a version of the NSGA-2 algorithm [51]. The parameters to execute the algorithm and the obtained results on a dataset of 200 customers are shown in Table 8. This setup was aimed to allow NGSA-2 to find the best values of each objective function $f_{1}$, $f_{2}$, $f_{3}$, and $f_{4}$. NGSA-2 showed the capability of searching for a Pareto front with more than 8000 solutions after more than 6 h of execution. NGSA-2 achieved solutions with the best values of $f_{2}$ = 0 and $f_{3}$ = 0, which are similar to those of the proposed algorithms; however, the proposed method was found it to be superior when looking for solutions for $f_{1}$ and $f_{4}$. The normalized distance of the closest solution to $z^{*}$ (fitness value) obtained by NGSA-2 was 0.122, which is inferior to that generated by the CP-based GA when using the similar searching mechanism. Although our results are not sufficient to conclude that the CP-based method is better than MOEA-2, the obtained Pareto front may contain lower quality solutions than those of the proposed method. During the search for the Pareto frontier, the search agents do not focus on achieving their goal as single objective optimization problem. The approach requires a significant computational overhead, and is thus difficult to adapt in a real-world environment. In addition, the user has no other choice, even if they only need to use one solution in reality, and other factors in the decision problem, such as user experience, do not contribute to this centralized search effort.

## 5. Conclusions

This study presents an adaptive method, MOP-VRP, to solve the urban shipment problem, based on CP and metaheuristics. The proposed model is a new variant of the VRP problem that combines different types of VRP and MOP, in which terrain and traffic conditions over time are integrated. We also designed three algorithms, GA, HGA, and TA, to solve the proposed model and compared their performance on a test dataset. Combining compromise programming and metaheuristics is a suitable approach to the MOP problem. However, once this approach is chosen, the decision-making process must respect compromise solutions instead of finding the Pareto frontier and assigning a solution based on higher-level information, unlike in other approaches, such as Pareto-dominance-based MOEA. In return, this approach allows a flexible design for many business scenarios. Traditional metaheuristics methods or hybrid versions can be smoothly applied with the CP-based system, although this system introduces weight parameters to the objective function. In practice, the selection of these values depends heavily on the decision maker’s experience and the business context for the use of the model, because re-executing the algorithm with large datasets multiple times involves a prohibitive computation cost. Therefore, it is recommended as an option when the decision maker does not have sources to indicate the preferences that happen more often in practice. The test results show that the combination of GA and local search in HGA is superior in improving the quality of the solution. The original version of GA may use trivial sampling points, but the nearest neighbor search can provide better genes to the next generation. This combination produces a high-quality solution without requiring an excessive computational cost, unlike the nearest neighbor search with a memory mechanism in TA. In our upcoming work, we will integrate the VRP model with integral logistics problems. The priority will be the improvement in the algorithm using recent advances in metaheuristics.

## Figures and Tables

**Table 1:** Research and corresponding objective functions.

Research	Objective Functions	VRP Types	Highlights	Drawbacks
Zhen et al., 2020 [9]	Minimize traveling time of all the vehicles.	MD-VRP, MT-VRP, VRP-TW	The proposed mixed integer linear programming can clearly describe the business. The proposed metaheuristics can provide optimal solutions on a large scale.	The model is not based on a realistic problem where the data is also randomly selected from the benchmark. The model does not involve several factors such as traffic conditions.
Babaee Tirkolaee et al., 2019 [10]	Minimize the sum of vehicle cost, traveling cost, penalty cost of soft time window.	MT-VRP, VRP-TW	A case study is investigated to evaluate the applicability of the proposed model in the real world. Many business conditions have been considered.	The business rules assume that the time and cost of a route is the same for all vehicles. This may not be guaranteed in other real-life applications. The designed solver can solve the problem in small and medium sizes that only offers near-optimal solutions compared to the CPLEX solver.
Alemany et al., 2018 [7]	Minimizing distribution cost and distance-based cost.	C-VRP, MP-VRP, MD-VRP	The model was developed from a realistic case study from an oil provider company.	Experiments are conducted on a small-scale dataset. Evaluations of the proposed method did not show its performance with different techniques.
Pan et al., 2021 [14]	Minimizing the traveling cost.	MT-VRP, VRP-TW	The routing solver was designed for a vending cafe company to replenish stocks for their geographically dispersed outlets. The proposed method can work on large-scale instances.	Authors simulate the experimented data by randomly creating data based on an existing dataset.
Ma et al., 2017 [15]	Minimizing traveling cost.	MD-VRP, VRP-TW	An improved ACO algorithm with some ideal to improve the search speed was introduced to solve the proposed problem.	The system considers only a single depot, which is not guaranteed in several applications. The research did not show the evaluations of the proposed algorithm using the existing methods.
Zhang et al., 2020 [16]	Minimize carbon emission.	MD-VRP	The research develops a new extension model of MD-VRP. The proposed algorithms can deal with large-scale datasets.	The proposed mathematical model and the heuristic algorithm provide better quality than the heuristic but with more computational cost.
Nucamendi-Guillén et al., 2021 [17]	Minimize the cost of transport and contracts.	CH-VRP, MD-VRP	The proposed model was obtained from a real-world business.	Business rules are simple. The designed metaheuristic was only tested with a small-scale dataset.
Li et al., 2020 [12]	Minimize completion time of vehicles.	MT-VRP, VRP-TW	The solver can be applied to some real-life problem instances. The proposed heuristic algorithm shows a better result than that of the CPLEX solver.	The model is simple and cannot be adapted to other businesses. The designer did not consider the concerns of different stakeholders in the system.
Shelbourne et al., 2017 [18]	Minimize the sum of total distance cost and total weighted tardiness.	VRP-TW	Proposed solvers based on heuristics were used to evaluate the performance on several datasets.	The optimization model was based on several assumptions that may not be applied to other situations

**Table 2:** System configuration for experiments.

Item	Info
CPU	Intel(R) Core (TM) i5-8350U CPU @ 1.70 GHz 1.90 GHz
RAM	Corsair Vengeance LPX 8 GB
Programming Platform	Java 8
Operating System	Windows 10

**Table 3:** Parameters used to conduct the experiments.

Parameter	GA	HGA	TA
Population size	1000	100	1
Crossover rate	0.9	0.9	None
Mutation rate	0.3	0.3	None
Selection rate	0.1	0.1	None
Stop condition	100	100	100
Neighborhood structure	None	Replace	Replace
Scanned Neighbors	None	1000	-
Tabu tenure	None	None	3

**Table 4:** Results obtained by solving the problem as separate single-objective problems.

i	$\;z_{i}^{*}$	$\;z_{i}^{worst}$
1	699.32	5045.18
2	0	701,979.5
3	0	12,137.6
4	1.35	9582.21

**Table 5:** Best results obtained by the proposed algorithms.

Algorithm	Solution Quality							Average Time (min)
	Average Fitness	Best Solution					Worst Fitness	
		Fitness	$f_{1}$	$f_{2}$	$f_{3}$	$f_{4}$		
TA	0.052	0.048	0.076	0.079	0.021	0.027	0.055	25.4
GA	0.073	0.068	0.041	0.096	0.013	0.057	0.076	5.35
HGA	0.050	0.045	0.047	0.068	0.016	0.034	0.053	12.6

**Table 6:** Traveling paths of 10 shippers to deliver 200 packages from 5 warehouses, as generated by HGA.

k	$O_{k}$	$f_{1}$	$f_{2}$	$f_{3}$	$f_{4}$
1	3-28-4-3-2-1-102-124-148-154-110-146-123-132-94-137-115-204-205-203-114-158	107.9532	8350.7	4.4	0.255
2	1-2-4-3-5-60-184-188-198-51-52-162-141-75-40-93-143-92-128-160-67-199-200-29-108-18-66-96-113-171-25-138-44-35-71-179-2-3-152-32	125.272	5687.2	57.3	141.555
3	5-4-2-202-126-151-147-107-142-156-190-193-127-100-30-145-112-165	96.75424	6272.4	1.9	−6.895
4	5-4-1-3-43-173-125-78-22-6-56-176-81-169-76	78.75142	973.15	0	−37.845
5	5-2-3-4-1-170-13-79-55-20-185-19-129-15-195-197-24-182-109-136-164-150-133-65-27-201-33-180-99-80-50	110.138	4980.6	0	−0.395
6	3-9-4-1-175-7-38-59	24.40988	149	0	−118.295
7	2-4-3-1-5-117-57-134-103-11-14-192-42-41-161-186-166-62-72-23-177-16-194-31-183-10-46-74-58-68	101.6263	7904.1	0	0.055
8	5-1-4-3-2-36-187-105-89-39-90-88-116-206-106-159-86-172-168-155-163-12-97	89.68427	3929.95	28.3	−0.845
9	5-4-3-2-1-178-101-149-181-130-84-82-48-8-191-49-157-189-21-34-77-120-153-91-174-53-131-63-69-37-85-118	87.14513	4240.15	92.3	22.205
10	5-1-2-4-3-87-47-83-73-111-26-119-70-121-64-17-122-167-61-140-104-98-45-54-139-144-196	84.42806	5860.5	14.4	0.205

**Table 7:** Best results obtained by the proposed algorithms.

Algorithm	HVC
TA	0.938
GA	0.885
HGA	0.941

**Table 8:** Results obtained on the tested dataset using NGSA-2.

Parameter/Criteria	Applied/Obtained by NGSA-2	Applied/Obtained by CP-Based GA
Population	10,000	1000
Stop Condition	1000	100
Crossover rate	0.8	0.9
Mutation rate	0.3	0.3
Average Execution Time (min)	~372	~5
Number of Solutions	~8837	1
$\mathrm{Best}\;\mathrm{found}\;f_{1}$	1422	699.32
$\mathrm{Best}\;\mathrm{found}\;f_{2}$	0	0
$\mathrm{Best}\;\mathrm{found}\;f_{3}$	0	0
$\mathrm{Best}\;\mathrm{found}\;f_{4}$	42	1.35
Best fitness value	0.122	0.0689

## Data Availability

Not applicable.
